# Fabrication and Characterization of Flexible Electrowetting on Dielectrics (EWOD) Microlens

**DOI:** 10.3390/mi5030432

**Published:** 2014-07-04

**Authors:** Chenhui Li, Hongrui Jiang

**Affiliations:** Department of Electrical and Computer Engineering, University of Wisconsin-Madison, Madison, WI 53707, USA; sam.lichenhui@gmail.com

**Keywords:** microlenses, microlens arrays, compound eye, tunable lenses, hydrogels

## Abstract

We present a flexible variable-focus converging microlens actuated by electrowetting on dielectric (EWOD). The microlens is made of two immiscible liquids and a soft polymer, polydimethylsiloxane (PDMS). Parylene intermediate layer is used to produce robust flexible electrode on PDMS. A low-temperature PDMS-compatible fabrication process has been developed to reduce the stress on the lens structure. The lens has been demonstrated to be able to conform to curved surfaces smoothly. The focal length of the microlens is 29–38 mm on a flat surface, and 31–41 mm on a curved surface, varying with the voltage applied. The resolving power of the microlens is 25.39 line pairs per mm by a 1951 United States Air Force (USAF) resolution chart and the lens aberrations are measured by a Shack-Hartmann wavefront sensor. The focal length behavior on a curved surface is discussed and for the current lens demonstrated the focal length is slightly longer on the curved surface as a result of the effect of the curved PDMS substrate.

## 1. Introduction

Liquid lenses do not require complicated mechanical systems to change their focal lengths, and they are widely used in photonics, display and biomedical systems [1–4]. Another rapidly-developing area in micro-optics is the fabrication of microlenses made on flexible polymer substrates [5,6], because microlens array on a curved substrate have some significant advantages over planar microlenses, including wider field of view [6,7], creating 3-D effect [8,9] and mimicking artificial compound eyes [10]. In virtue of these developments, emerging liquid-based variable-focus microlenses have become important components in modern miniaturized optical systems. Benefitting from the quick response, the low power consumption, and the robustness under voltage cycling, liquid microlenses based on electrowetting on dielectric (EWOD) have drawn much attention [1,11]. However, traditional EWOD microlenses are fabricated on rigid materials like glass, silicon and polyethylene terephthalate, and are consequently not compatible with curved surfaces.

We have previously reported a flexible EWOD liquid lens design [12], in which a water droplet is covered by silicone oil and the water droplet has to turn concave to form a converging lens. Therefore, the electrode, dielectric and hydrophobic layers were deposited on the sidewall of a polymer chamber to change the water surface from convex to concave shape and the water-oil interface is pinned on the sidewall of the chamber. This design raises two problems: (1) When the lens is wrapped onto a curved surface, the sidewall will be stretched and it is difficult to determine how the water-oil interface will be distorted; (2) The electrode and hydrophobic coating on the sidewall cannot be easily patterned. In addition, the indium tin oxide (ITO) electrode is fragile due to the weak adhesion of ITO on polydimethylsiloxane (PDMS), a soft polymer.

Here, we present a flexible EWOD microlens made of PDMS with improved lens design and robust flexible electrodes. We also discuss the focal length of the microlens on a spherical surface in comparison to that of the same lens on a flat surface. The center of the lens is a silicone oil droplet covered by surrounding water. The thin flexible PDMS substrate is compatible with curved surfaces and the effect of chamber sidewall distortion on the water-oil interface is reduced. The modified low-temperature fabrication process is also reported. A parylene C layer is coated directly onto PDMS to promote aluminum-PDMS bonding and to reduce the problem associated with the porosity of PDMS. The focal length is measured on both flat and curved substrates. When the applied voltage increases up to 100 V, the focal length changes from 38 mm to 29 mm on a flat substrate and it is slightly longer on a curved substrate (*f*: 41–31 mm). The Zernike coefficients of the lens were measured by a Shack-Hartmann wavefront sensor.

## 2. Mechanism and Fabrication

### 2.1. Mechanism

**Figure 1:**
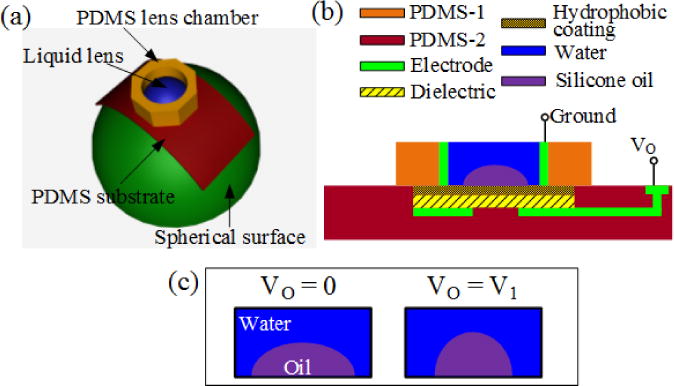
(**a**) 3D schematic of a flexible liquid lens on a hemisphere. The lens structure is made of a soft polymer, polydimethylsiloxane (PDMS). The thin flexible PDMS substrate can be wrapped onto curved surfaces; (**b**) aross-section schematic of the microlens. The PDMS substrate is deposited with an electrode, a dielectric layer, and a hydrophobic coating subsequently, and the PDMS chamberf is coated with another electrode. The liquid lens is the liquid-to-liquid interface formed by a silicone oil droplet and its surrounding water. The voltage applied on the electrodes modifies the effective surface energy, and thus changes the radius of curvature of the water-oil interface; (**c**) at zero or low voltage, the silicone oil droplet spreads out on the substrate due to the hydrophobic coating. As the voltage increases, the substrate surface turns more hydrophilic and the water squeezes the oil droplet to a shape with smaller radius of curvature.

Figure 1a shows a 3D schematic of a flexible microlens wrapped onto a spherical surface. The lens substrate and lens chamber are both made of PDMS, which is transparent, flexible, and bio-compatible. The substrate is designed to be much thinner than the chamber. Therefore, the lens can be easily wrapped onto curved surfaces and the stress on the chamber is significantly reduced [6]. Figure 1b illustrates the cross-section of a flexible EWOD microlens. A silicone oil droplet (refractive index *n*_1_ = 1.47) is placed at the center of the chamber and it is covered by water (refractive index *n*_2_ = 1.33). The electrode, dielectric and hydrophobic layers are sequentially coated on the PDMS substrate and they were patterned by photolithography techniques. The water is in contact with another electrode on the chamber. The applied voltage between the two electrodes controls the surface energy on the substrate and therefore varies the radius of curvature of the water-oil interface [1]. Figure 1c shows the change of the water-oil interface when a voltage is applied. At low voltages, the substrate is hydrophobic and the oil droplet spreads on it, forming a small contact angle. As the voltage increases, the substrate turns relatively hydrophilic and water squeezes the oil droplet to a more convex shape, which has a larger contact angle with the substrate. The varying contact angle (*θ*) of the lens can be expressed by Equation (1) [11].

(1) $$\cos(\theta )=\cos(\theta_{0})+\frac{\varepsilon }{2d\gamma_{12}}V_{0}^{2}$$

where *θ*_0_ is the contact angle at *V*_0_ = 0, *ε* is the dielectric constant of the dielectric layer, *d* is its thickness, *γ*_12_ is the water-oil interfacial tension and *V*_0_ is the applied voltage.

### 2.2. Fabrication

It is difficult to create strong permanent bonding between metal and PDMS surface due to the low surface energy of PDMS [13] and the different degrees of thermal expansion of the metal and PDMS layers during the metal deposition process [14]. Therefore, surface treatment is necessary to increase the surface energy of PDMS; otherwise, the weak adhesion between electrodes and PDMS substrate often cause device failure during the following electrode patterning or substrate bending. A few approaches have been tried out and proved to significantly strengthen the electrode-PDMS bonding:

1. oxygen plasma treatment increases the bond strength by activating layers of cross-linked PDMS in oxygen plasma; surface oxidation is believed to expose silanol groups (OH) at the surface of the PDMS layers that when brought together form covalent siloxane bonds (Si–O–Si) [15,16];
2. polyimide intermediate layer, which is widely used in flexible electronic devices [17,18];
3. parylene C coating, on which metal layers can be deposited and patterned [19,20]. Besides promoting electrode-PDMS adhesion, parylene C layer also improves the overall device robustness. Parylene has attractive properties, such as optical transparency, pinhole-free conformal deposition process [21], chemical and biological inertness, good mechanical strength (Young’s modulus of 3.1–4.75 GPa and tensile strength of 40–110 MPa) [21–23], low permeability to moisture and gases [24] and low intrinsic film stress. The parylene C coating addresses the porosity problem of PDMS which could cause bubble formation, sample evaporation and absorption of organic solvents and small hydrophobic molecules from solution [25]. Therefore, deposition of parylene C coatings onto PDMS film is used as the surface treatment method in the fabrication process.

**Figure 2:**
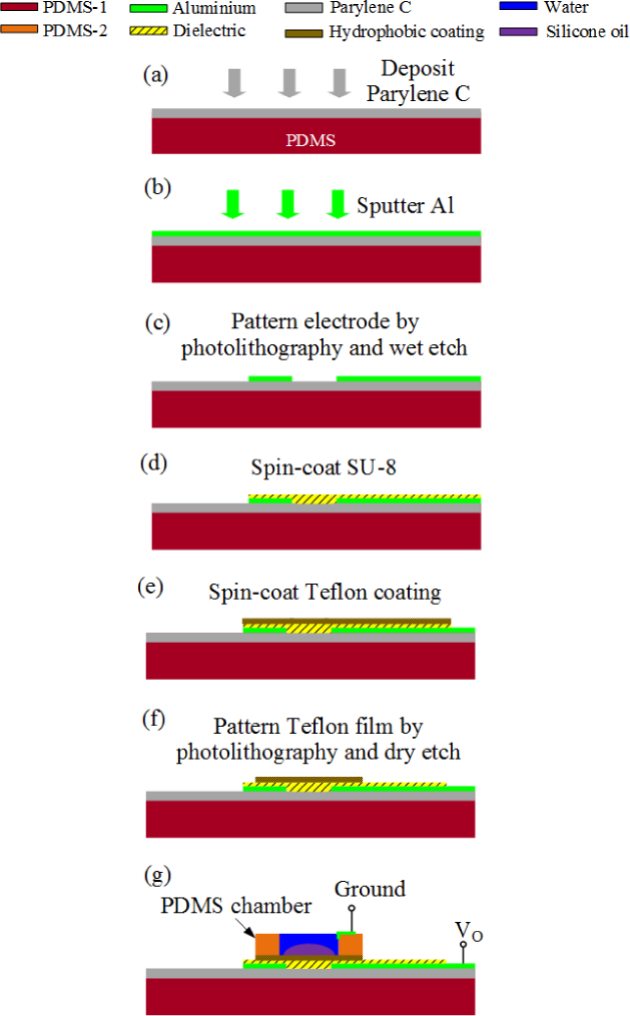
Fabrication process of the flexible electrowetting on dielectric (EWOD) microlens. (**a**) Deposit parylene C layer (~2 μm) on PDMS substrate. Parylene strengthens the boding between PDMS and electrodes; (**b**) and (**c**) sputter aluminum electrode (~200 nm) and pattern it by photolithography and wet etch; (**d**) spin-coat SU-8 dielectric layer (~2.4 μm) and post-bake it at 120 °C for 5 h; (**e**) spin-coat Teflon hydrophobic thin film (~400 nm) and post-bake it at 120 °C for 10 h to remove the solvent; (**f**) sattern the Teflon coating with photolithography and reactive ion etching; (**g**) sond a PDMS chamber onto the substrate using a flexible, UV-cure adhesive.

The fabrication process starts with preparing the PDMS substrate. A clean microscope slide is coated with a hydrophobic layer, trichloro(octadecyl)silane (Sigma-Aldrich Co., Saint Louis, MO, USA), to facilitate the separation of PDMS film from the microscope slide later on. The microscope slide is then spin-coated with PDMS prepolymer at 1000 rpm for 60 s and then baked on a hotplate at 70 °C for 4 h to cure the prepolymer. Figure 2 illustrates subsequent fabrication processes on the PDMS substrate. In Figure 2a, a thin parylene C film (~2 μm) is deposited on the PDMS substrate to strengthen electrode adhesion and to mitigate the porosity problem of PDMS as discussed above. In Figure 2b, aluminum film (~200 nm) is sputtered on the parylene-coated PDMS in a sputtering system (Discovery 24, Denton Vacuum, Moorestown, NJ, USA). In Figure 2c, the aluminum electrode is photopatterned with photoresist AZ4620 (Clariant Corporation, Somerville, NJ, USA) and then wet-etched in a commercially prepared defreckling aluminum etchant for 5 min. SU-8 is used as the dielectric material, because it has high dielectric constant (~3) and can be deposited by spin-coating method. In Figure 2d, the substrate is spin-coated with SU-8 prepolymer solution (SU-8 2002, MicroChem Corp., Newton, MA, USA) at 500 rpm for 8 s and then at 2000 rpm for 30 s. Next, the SU-8 undergoes UV exposure at 20 mW for 40 s, followed by hard bake at 120 °C for 5 h, forming a ~2.4 μm SU-8 film. In Figure 2e, the SU-8 dielectric layer is spin-coated with Teflon solution (Teflon^®^ AF 1600, DuPont, Wilmington, DE, USA) at 1000 rpm for 30 s and then baked at 120 °C for 10 h. To avoid damages to the PDMS, the baking temperature is much lower than that used in standard procedure (up to 330 °C) [26], and therefore the baking time is extended to remove the solvents thoroughly. The Teflon film formed (~400 nm thick) is hydrophobic, smooth and chemical-inert. In Figure 2f, the Teflon film is photopatterned with photoresist AZ4620 and then etched by oxygen plasma at 200 W for 2 min in a reactive ion etching (RIE) chamber (Unaxis 790, Plasma-Therm, St Petersburg, FL, USA). In Figure 2g, a PDMS chamber is bonded to the substrate by an acrylic, low durometer, flexible, UV-cure adhesive (Loctite 3108, Henkel Corp., Rocky Hill, CT, USA). The adhesive is cured under 30 mW UV radiance for 20 s to form a rubbery bonding. The chamber is first filled with water and then the silicone oil droplet is squeezed to the center by a syringe pipette with a sharp tip.

**Figure 3:**
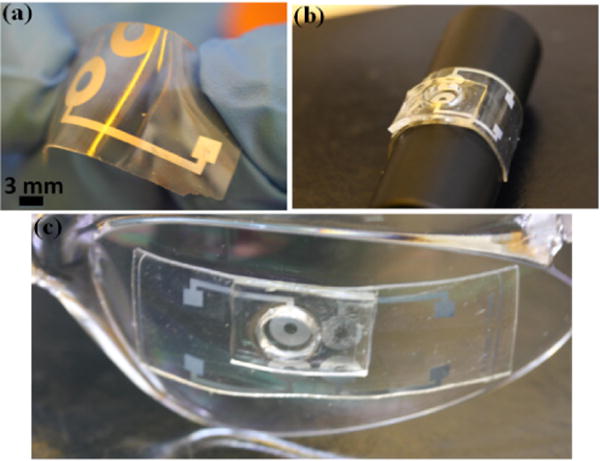
Demonstration of the flexible PDMS lens structure: (**a**) Bending a PDMS substrate (already coated with Al electrode, SU-8 dielectric layer and Teflon coating); (**b**) a flexible EWOD lens wrapped on a cylinder; (**c**) a flexible EWOD lens bonded with a protection glass, which has almost zero optical power.

Figure 3 demonstrates the flexible EWOD microlens and its compatibility with curved surfaces. Figure 3a shows the thin PDMS substrate after it is deposited with aluminum electrode, SU-8 dielectric and Teflon hydrophobic coating. Figure 3b,c shows the EWOD lens wrapped onto a cylinder and an eyeglass, respectively. The aperture (circular opening on the substrate) is 2 mm in diameter and the diameter of the PDMS chamber is around 8 mm. The PDMS lens structure exhibits excellent flexibility and transparency.

## 3. Results and Discussion

### 3.1. Experiment Results

**Figure 4:**
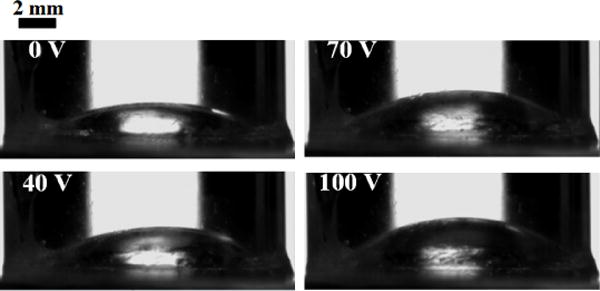
Shape change of oil droplet with increasing voltage. As the applied voltage increases, the oil droplet is squeezed by the surrounding waters and bulges up into a shape with smaller radius of curvature.

The shape change of the droplet within the microlens is monitored by a goniometer (OCA 15+, DataPhysics Instruments Inc., Filderstadt, Germany). Figure 4 shows the cross-section images of the silicone oil droplet when the applied voltage, 500 Hz Alternating Current (AC), varies between 0 and 100 V. The volume of the oil droplet is 54 μL. When the voltage increases, the effective surface energy on the substrate increases, and thus the substrate turns more hydrophilic. Therefore, the water squeezes the oil droplet, reducing the radius of curvature (*R*) of the oil-water interface. The focal length is thus to decrease with increasing voltages.

**Figure 5:**
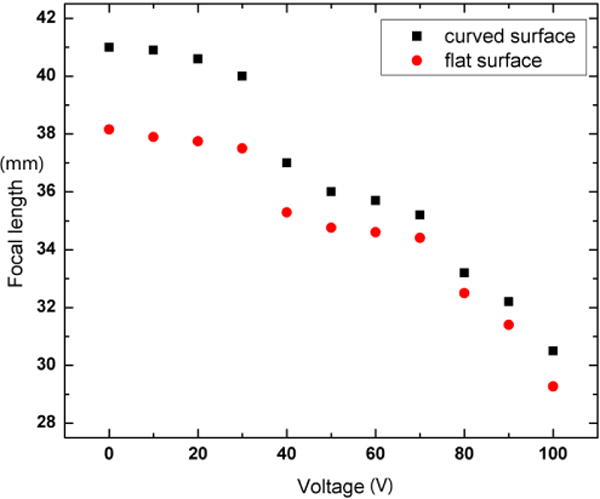
Focal length versus applied voltage. The curved surface is the protection glass shown in Figure 3c and it has no optical power. On the curved surface, the lens has a slightly longer focal length than that on a flat surface.

The focal length of the lens was measured on both a flat and a curved transparent surface. The curved surface, which is the eyeglass in Figure 3c, has zero optical power. Figure 5 shows the focal length *f* versus the applied voltage. On both substrates, the focal length varies in a similar trend with increasing voltages; however the lens has a slightly longer focal on the curved surface. The possible source of the focal length change will be discussed in Section 3.2. The resolving power of the lens is measured by imaging a 1951 United States Air Force (USAF) resolution test chart and the smallest features to resolve were 25.39 line pairs per mm.

### 3.2. Discussion of Focal Length Change

As shown in Figure 5, the liquid microlens exhibits slightly longer focal length on a curved surface than that on a flat surface. The change could have two sources: (1) the oil droplet forms a different shape on the curved PDMS substrate; (2) the curved PDMS substrate changes the overall optical power of the system. Based on the current fabrication process, the microlens cannot be wrapped onto a curved substrate that has comparable radius of curvature to that of the oil droplet. In other words, the curved substrate has a much larger radius compared to the liquid lens. Therefore, it is reasonable to assume that the shape of the oil droplet did not change much when the microlens was wrapped onto the curved surface.

**Figure 6:**
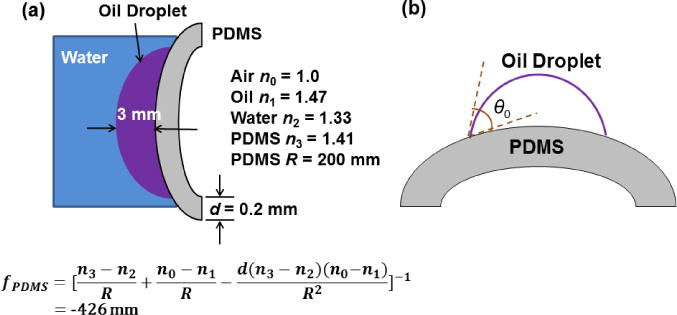
(**a**) Schematic of the overall optical system when the liquid lens is on a curved surface. The PDMS substrate forms a diverging lens and its focal length is around −426 mm; (**b**) contact angle of an oil droplet on curved PDMS substrate.

Next, we consider the effect of the optical power of the curved PDMS substrate. Figure 6a illustrates the optical system when the microlens is on curved surface. The water-oil interface has a focal length *f*_1_ = 38 mm. The curved PDMS substrate functions as a diverging lens with focal length *f*_2_ = −426 mm. By lensmakers’ formula, the overall focal length is calculated by:

(2) $$\frac{1}{f}=\frac{1}{f_{1}}+\frac{1}{f_{2}}-\frac{d}{n_{1}f_{1}f_{2}}$$

where *d* ≈ 3 mm and *n*_1_ = 1.47 (oil’s refractive index). Therefore, the estimated overall focal length is 41.5 mm, which is close to the experiment result, 41 mm. To sum up, for the current microlens device the curved PDMS substrate is the dominant cause of the focal length change and the diverging lens formed by it makes the system focal length slightly longer than that on a flat substrate.

In the future, with improved fabrication process, the lens could be wrapped onto a convex substrate with much shorter radius of curvature than that of the current one. Under such circumstances, the shape change of the oil droplet must be taken into consideration. Under ideal conditions, such as that the liquid droplet is deposited on the substrate symmetrically and that the hysteresis in contact angle is neglected, the contact angle between substrate-liquid surface tangent and the liquid-liquid surface tangent is the same as the *θ*_0_ in Equation (1) [27], as shown in Figure 6b. As a result, the focal length of the liquid lens will be shorter on the curved substrate, excluding the effect of the curved PDMS substrate.

## 4. Conclusions

In summary, we have demonstrated a new design of flexible EWOD microlens which is made of soft flexible PDMS structure. The process to fabricate robust electrodes on PDMS thin substrate is also introduced. Parylene C thin film is used as an intermediate layer to strengthen the bonding between PDMS and aluminum electrode and to address the porosity problem of PDMS. The liquid lens is formed by a silicone oil droplet and the water covering it. All the functioning layers implementing EWOD mechanism are deposited on the substrate. Therefore, when a lens is wrapped onto a curved surface, the effect of stress on the water-oil interface is reduced as the peripherals of the oil droplet are pinned on the substrate. The microlens is a converging lens at any voltage. When the applied voltage increases, the water squeezes the oil droplet, and thus both the radius of curvature of the droplet and the focal length decreases. The focal length is 29–38 mm on a flat surface and 31–41 mm on a curved surface. The curved PDMS substrate forms a diverging lens and makes the overall focal length slightly longer on the curved surface. The aberration of the lens is measured by a Shack-Hartmann wavefront sensor and its Zernike coefficients are reported. In the future, the driving voltage of the lens can be further reduced by exploring dielectric materials which have high dielectric constants and are compatible with our flexible polymer structure. The fabrication process can be improved to make the lens structure compatible with a more curved substrate. A contact lens integrated with a flexible low-voltage EWOD microlens provides a better solution for presbyopia correction. Meanwhile, EWOD microlens array can also be fabricated by the current fabrication process and it can be formed on a spherical surface to provide a micro-optical system with very wide field of view [6].
